# Early Proliferation Does Not Prevent the Loss of Oligodendrocyte Progenitor Cells during the Chronic Phase of Secondary Degeneration in a CNS White Matter Tract

**DOI:** 10.1371/journal.pone.0065710

**Published:** 2013-06-11

**Authors:** Sophie C. Payne, Carole A. Bartlett, Donna L. Savigni, Alan R. Harvey, Sarah A. Dunlop, Melinda Fitzgerald

**Affiliations:** 1 Experimental and Regenerative Neurosciences, School of Animal Biology, The University of Western Australia, Crawley, Western Australia, Australia; 2 Experimental and Regenerative Neurosciences, School of Anatomy, Physiology and Human Biology, The University of Western Australia, Crawley, Western Australia, Australia; Hospital Nacional de Parapléjicos - SESCAM, Spain

## Abstract

Partial injury to the central nervous system (CNS) is exacerbated by additional loss of neurons and glia *via* toxic events known as secondary degeneration. Using partial transection of the rat optic nerve (ON) as a model, we have previously shown that myelin decompaction persists during secondary degeneration. Failure to repair myelin abnormalities during secondary degeneration may be attributed to insufficient OPC proliferation and/or differentiation to compensate for loss of oligodendrocyte lineage cells (oligodendroglia). Following partial ON transection, we found that sub-populations of oligodendroglia and other olig2+ glia were differentially influenced by injury. A high proportion of NG2+/olig2–, NG2+/olig2+ and CC1−/olig2+ cells proliferated (Ki67+) at 3 days, prior to the onset of death (TUNEL+) at 7 days, suggesting injury-related cues triggered proliferation rather than early loss of oligodendroglia. Despite this, a high proportion (20%) of the NG2+/olig2+ OPCs were TUNEL+ at 3 months, and numbers remained chronically lower, indicating that proliferation of these cells was insufficient to maintain population numbers. There was significant death of NG2+/olig2– and NG2−/olig2+ cells at 7 days, however population densities remained stable, suggesting proliferation was sufficient to sustain cell numbers. Relatively few TUNEL+/CC1+ cells were detected at 7 days, and no change in density indicated that mature CC1+ oligodendrocytes were resistant to secondary degeneration *in vivo*. Mature CC1+/olig2– oligodendrocyte density increased at 3 days, reflecting early oligogenesis, while the appearance of shortened myelin internodes at 3 months suggested remyelination. Taken together, chronic OPC decreases may contribute to the persistent myelin abnormalities and functional loss seen in ON during secondary degeneration.

## Introduction

Partial injuries to the CNS are a common consequence of head or spinal cord injury trauma that involve the loss of neural tissue directly damaged by the focal insult, and the additional widespread loss of residual tissue via secondary degeneration. The loss of residual neurons and glia results from a cascade of destructive secondary events, including excitotoxicity and calcium ion overload, mitochondrial dysfunction and depletion of adenosine-triphosphate (ATP), oxidative stress and cell death [1], [2]. Loss of oligodendrocytes and demyelination of intact axons is a key pathology that causes functional deficits [3] following CNS injury [4] and in multiple sclerosis [5]. *In vitro* experiments indicate that oligodendroglia are vulnerable to excitotoxic insult and oxidative stress depending upon their maturation state [6], [7]. However, oligodendroglia sensitivity and responses to injury-related cues and secondary degenerative events *in vivo* are unknown. Partial transection of the ON is a model in which the primary injury (dorsal ON) is spatially segregated from the area of secondary degeneration (ventral ON) [8], [9]. Following partial ON transection, we have previously demonstrated that chronic decompaction of myelin is a feature of secondary degeneration that persists for as long as 6 months, and is associated with progressive loss of visual function [10], [11].

The persistence of myelin abnormalities and associated loss of function during chronic secondary degeneration occurs despite the potential for remyelination [12], [13]. Myelin can be reinvested to naked axons, restoring saltatory conduction [14] and function [15]. Remyelination is mediated by oligodendrocyte progenitor cells (OPCs) [16] that express the chondroitin sulphate proteoglycan NG2 [17], [18]. NG2+ cells are a heterogeneous population [19] that have a neuromodulatory role in nerve signaling at the nodes of Ranvier in white matter [20] and are stem cell - like in that they have the capacity to differentiate into astrocytes or neurons [21]. It has been shown *in vivo* that NG2+ cells function as OPCs that respond to demyelination by proliferating, migrating to the injury site [22], [23] and differentiating into mature myelinating oligodendrocytes [24]. We hypothesise that myelin decompaction observed during secondary degeneration is related to disruption in OPC proliferation and/or differentiation.

The phenotypic and transcriptional changes OPCs undergo during differentiation allow the identification of these cells at various stages of maturity [13], [14]. To enable quantification of sub-populations of oligodendroglia and olig2+ glia in ON vulnerable to secondary degeneration, we immunohistochemically detected the immature marker NG2 and mature marker CC1, combined with olig2. Specifically, we assessed whether OPC proliferation was sufficient to maintain oligodendroglia and other olig2+ glia during secondary degeneration by comparing proliferation (Ki67) and death (TUNEL) of these populations. Furthermore, axons were anterogradely labelled with the neurotracer CTB, anti-Caspr to identify paranodes and anti-Nav1.6 to identify sodium channels at the nodes, allowing determination of myelin internode length as an indicator of remyelination. Our data showed that OPCs were vulnerable to injury, and the chronic decrease in numbers of this population was not alleviated by OPC proliferation.

## Results

### Oligodendroglia Populations in Control Optic Nerve

**Figure 1:**
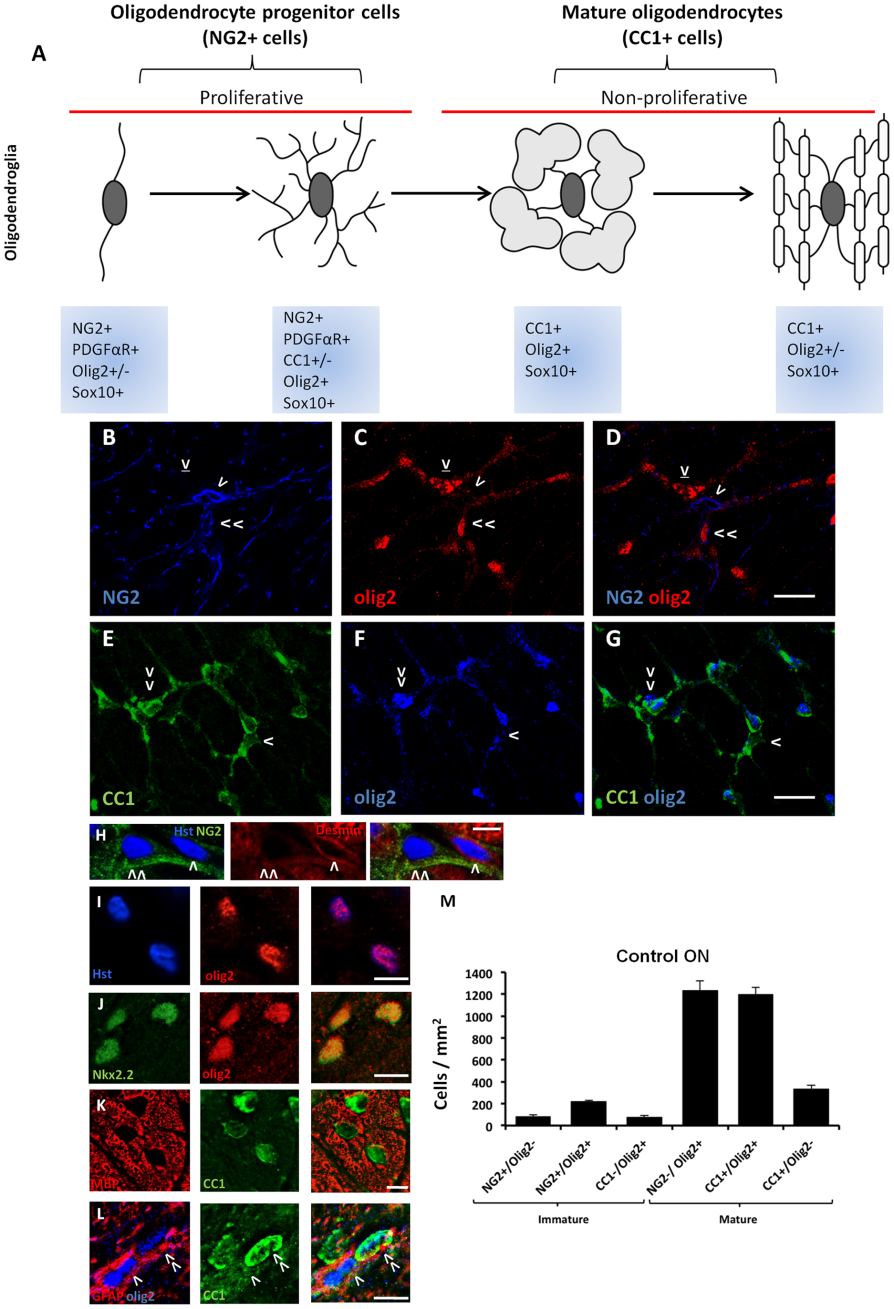
Oligodendroglia subpopulations of varying maturity in adult control ON. A: Schematic diagram illustrates changes in the expression of NG2 and CC1 markers, and olig2 transcription factor across oligodendroglia subpopulations [24]. Oligodendroglia and olig2+ glia were identified with combinations of antibodies to NG2 (B, D) and olig2 (C, D), or CC1 (E, G) and olig2 (F, G). D: Cells indicated are NG2+/olig2– (>), NG2+/olig2+ (>>) or NG2−/olig2+ (>l). G: Cells indicated are CC1+/olig2– (>) or CC1+/olig2+ (>>). H: Desmin+ cells (>) were not NG2+ (>>). Olig2 staining colocalises with Hoechst nuclear stain (I) and Nkx2.2+ nuclei (J). K: MBP+ myelin surrounds CC1+ oligodendrocyte somata. L: GFAP immunoreactivity surrounds some olig2+ nuclei (example >) but does not colocalise with CC1 (>>). M: Quantification of immunopositive oligodendroglia in control ON was expressed as the mean density of cells per mm^2^ ± S.E. Scale bars: B–G: 20 µm, H–L: 10 µm.

Oligodendroglia at different stages of maturity can be quantified using cell specific markers and transcription factors (Fig. 1A). To identify immature oligodendroglia, ON sections were immunohistochemically labelled with NG2, expressed by OPCs [18], [25] +/− olig2, a transcription factor expressed across the oligodendrocyte lineage [26], [27] (Fig. 1A–D). NG2 expression *in vivo* has been detected in macrophages [28], [29], astrocytes [30], microglia/monocytes [31] and pericytes [32] following CNS injury. We therefore conducted pilot experiments and confirmed that NG2+ cells did not co-localise with GFAP+ astrocytes, IBA1+ microglia/macrophages or desmin+ pericytes in control or injured ON (n = 3/group, representative desmin negative NG2 cell Fig.1H). NG2+ cells were also distinguished as OPCs based on morphology, and were only included if they featured small cell bodies with multiple processes (Fig. 1B indicated by > or >>) that were distinct from the large profiles of macrophages [28]. Furthermore, NG2+ cells were not associated with blood vessels and were therefore unlikely to be pericytes [33]. As such, in ON vulnerable to secondary degeneration, the majority of NG2+ cells are considered to be OPCs. Furthermore, the co-expression of NG2 and olig2 confirms that NG2+/olig2+ cells are OPCs [34]. Olig2 expression was predominantly nuclear as evidenced by colocalisation with the nuclear stain Hoechst (Fig. 1I). Nkx2.2 immunoreactivity was only observed co-localised with olig2+ nuclei in control ON and at 3 days after injury (Fig. 1J), although not all olig2+ nuclei expressed Nkx2.2. Use of PDGFαR together with NG2 has also been used to identify OPCs [35], however olig2 was used in preference to PDGFαR to allow identification of maturing OPC sub-populations [36].

Mature oligodendroglia were identified with anti-CC1, specific to the mature oligodendrocyte cell body marker adenomatous polyposis coli (APC) [37] and olig2 (Fig. 1A, E–G). Mature myelinating CC1+ cell somata were surrounded by MBP+ myelin (Fig. 1K), but did not colocalise with GFAP at any time point after injury (n = 3/group, Fig. 1L indicated by >>), in line with previous studies [38], [39].

Immunopositive cells were quantified in ventral ON, in the section encompassing the dorsal injury site. Several immunohistochemically distinct oligodendroglia populations were quantified as being immature: NG2+/olig2–, NG2+/olig2+, or mature: CC1+/olig2+ and CC1+/olig2– (Fig. 1A–G). We additionally quantified olig2+ cells that were negative for oligodendroglial markers (CC1, NG2), these being CC1−/olig2+ and NG2−/olig2+ cells. Olig2 has been shown to be upregulated in reactive astrocytes following injury [40] and pilot colocalisation experiments using antibodies to GFAP indicated that some CC1−/olig2+ cells were astrocytes (Fig. 1L indicated by >), in line with previous reports [27]. In control ON, the ratio of NG2+/olig2+ progenitor cells compared to mature CC1+ oligodendrocytes is approximately 1: 7 (Fig. 1M). The slightly higher density of NG2+/olig2+ cells compared to CC1−/olig2+ cells in control ON (Fig. 1M) may be due to the presence of a small population of NG2+/olig2+/CC1+ cells. While NG2 has been shown to colocalise with mature oligodendrocyte markers, numbers of these cells are generally low in both control tissue and following demyelinating injury [30], [41], [42], [43]. The NG2+/olig2+/CC1+ population is thought to represent a brief transitional stage [42] and this population was not directly quantified in the current study.

### Early Oligodendroglia Proliferation during Secondary Degeneration

**Figure 2:**
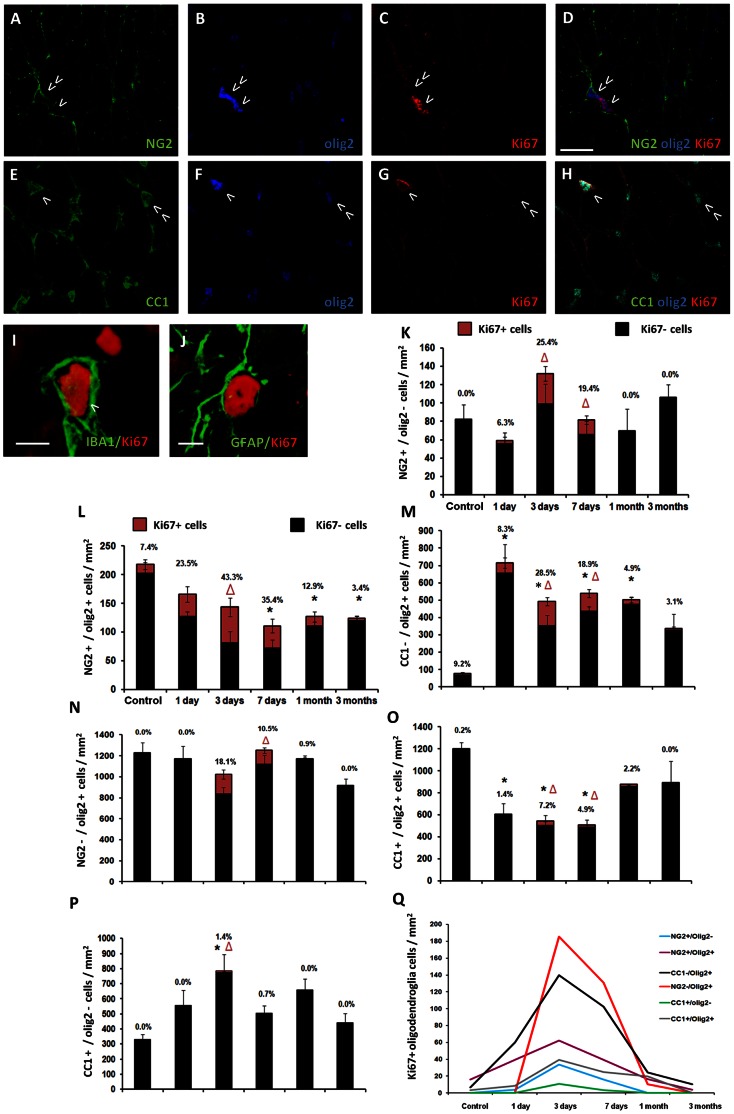
Proliferation of oligodendroglia subpopulations following partial ON transection. Oligodendroglia and other olig2+ glia were identified with antibodies to NG2 (A), olig2 (B) and Ki67 (C), or with CC1 (E), olig2 (F) and Ki67 (G). D: Cells indicated are Ki67+/NG2+/olig2+ (>) and Ki67−/NG2+/olig2+ (>>). H: Cells indicated are Ki67+/CC1+/olig2+ (>) and Ki67−/CC1+/olig2+ (>>). Proliferating Ki67+/IBA1+ cells (I, indicated by >) and to a lesser extent Ki67+/GFAP+ cells (J) were observed after injury; representative examples at 3 days shown. K–P: Quantification of the mean density ± S.E of oligodendroglia and other olig2+ glia populations following partial transection. Densities of Ki67– cells are represented by black bars while densities of Ki67+ cells are represented by red bars and differences from control indicated by Δ(p≤0.05). Overall differences in total density (combined Ki67+ and Ki67– values) compared to control are indicated by *(p≤0.05). Q: Summary graph of Ki67+ mean densities of all oligodendroglia and other olig2+ glia subpopulations. Scale bar A–H: 20 µm, I–J: 10 µm.

OPCs are the major cycling cells of the adult (undamaged) spinal cord, cerebral cortex, corpus callosum and hippocampus [41]. In control ON, we used the proliferation marker Ki67 [44] to show that 90.1% of Ki67+ cells were NG2+/olig2+ (Table 1, Fig. 2A–D) and 50.0% were also CC1−/olig2+ (Table 1, Fig. 2E–H), reflecting the overlap of these populations. Despite the apparent difference in percentages, there was no significant difference between the density of Ki67+/NG2+/olig2+ OPCs (16.08+8.12 cells/mm^2^) and Ki67+/CC1−/olig2+ glia (6.87+4.95 cells/mm^2^, p = 0.361). No proliferating NG2+/olig2– cells were detected in control ON (Table 1; Fig. 2I), suggesting that not all NG2+ cells were involved in cell cycling in the CNS, rather only OPCs co-expressing NG2 and olig2.

**Table 1:** Proportion of proliferating (Ki67+) cells that are immature oligodendroglia/CC1−/olig2+ glia.

	Proportion of Ki67+ cells that are immature oligodendroglia/CC1−/olig2+ glia		
	*NG2+/olig2–*	*NG2+/olig2+*	*CC1−/olig2+*
**Control**	0	90.1	50.0
**1 day**	5.1	53.9	49.9
**3 days**	4.1	7.6	24.6
**7 days**	5.2	12.9	37.3
**1 month**	0	42.1	40.9
**3 months**	0	71.9	83.8

Data are expressed as percentages of the means ± SEM of data presented in Figs. 2K–M. Note that values do not sum to 100% due to over-lapping populations.

It is well established that OPCs proliferate in response to CNS injury [45], [46]. Here, we assessed the proportion of the population vulnerable to secondary degeneration that was undergoing proliferation, so as to determine whether these proportions were sufficient to maintain oligodendroglia numbers (Fig. 2). Following partial ON transection, most Ki67+ cells were detected during the first week following injury (3 days: p<0.0001, 816.78±149.41 cells/mm^2^; 7 days: p = 0.073, 304.29±63.09 cells/mm^2^). The proportion of Ki67+ cells that were immature oligodendroglia/CC1−/olig2+ glia was 36.3% at 3 days and 55.4% at 7 days (summed from Table 1); the remainder of Ki67+ cells were predominantly microglia/macrophages and to a lesser extent, astrocytes (representative images Fig. 2I, J) [45]. With regards to immature oligodendrocytes, a high proportion of NG2+/olig2– cells were Ki67+ at 3 and 7 days, but no Ki67+ cells of this population were detected after this time point (Fig. 2K). The total density of NG2+/olig2– cells remained stable and similar to control at all time-points following injury (p>0.05; Fig. 2K). NG2+/olig2+ cells had the highest proportion of Ki67+ cells and this was sustained from 1 day until 1 month, although the proportion of NG2+/olig2+ cells proliferating at 3 months was lower than in control ON (Fig. 2L). Correspondingly, the total NG2+/olig2+ cell density remained similar to control at 1–3 days, but there were significant sustained decreases from 7 days to 3 months (Fig. 2L). The decrease in NG2+/olig2+ cell density at 3 months was not due to ON swelling [10], [11] as the total numbers of these cells in ventral ON also significantly decreased from control (p = 0.0014).

While a high proportion of CC1−/olig2+ cells were Ki67+ from 1 day until 1 month, reflecting overlap with the NG2+/olig2+ population (Fig. 1A, 2M, Q), the total density of CC1−/olig2+ cells dramatically increased at 1 day (p<0.0001) and remained significantly higher than control until 1 month (p<0.05; Fig. 2M). Previous studies have confirmed that olig2 is up-regulated in cells negative for NG2 and CC1, but positive for Sox10. It was suggested that such NG2−/CC1+/Sox10+ cells were either a separate precursor population within the oligodendroglia lineage, or were astrocytes, given that a small proportion of these cells colocalised with GFAP [27], [34]. Given our observation of CC1−/olig2+/GFAP+ cells (Fig. 1L) it is likely that the increase in CC1−/olig2+ at 1 day (Fig. 2M) reflects the up-regulation of olig2 in reactive astrocytes [40] (Fig. 2I). Ki67+/NG2−/olig2+ cells were detected only during the acute time period of 3–7 days after injury, with virtually none of these cells seen at any other time point (Fig. 2N). The total NG2−/olig2+ cell density was unchanged following injury (p>0.05; Fig. 2N).

With regards to mature oligodendrocytes, CC1+ cells are thought to be non-proliferative [47], [48]. Surprisingly however, we found that a modest proportion of the CC1+/olig2+ and CC1+/olig2– cell population expressed the proliferation marker Ki67 at 3 and 7 days following partial ON transection (Fig. 2H, O, P). The total density of CC1+/olig2+ cells rapidly decreased at 1 and 3 days; however, this loss was counter-balanced by a significant increase in the density of more mature CC1+/olig2– oligodendrocytes at 3 days (p = 0.041; Fig. 2P). Such changes perhaps suggest that at least a proportion of CC1+/olig2+ cells have differentiated into the more mature CC1+/olig2– oligodendrocytes.

### OPCs were Vulnerable to Secondary Degeneration *in vivo*, While Mature Oligodendrocytes were Resistant

**Figure 3:**
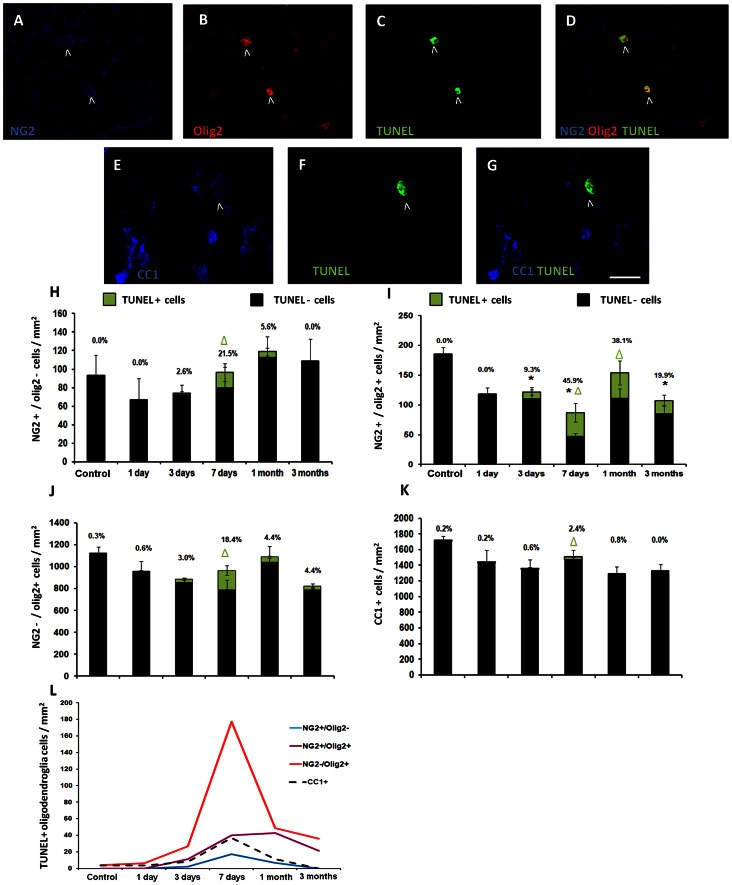
Death of oligodendroglia subpopulations following partial ON transection. Dying oligodendroglia and other olig2+ glia were identified with antibodies to NG2 (A), olig2 (B) and TUNEL (C), or with CC1 (E) and TUNEL (F). D: Cells indicated are TUNEL+/NG2+/olig2+ cells (>). G: The cell indicated is TUNEL+/CC1+ (>). Scale bar A–G: 20 µm. H–K: Quantification of the mean ± S.E density of oligodendroglia and other olig2+ glia following partial transection. Densities of TUNEL- cells are represented by the black bars while densities of TUNEL+ cells are represented by green bars and differences indicated by Δ(p≤0.05). Overall differences in total density (combined TUNEL+ and Ki67– values) compared to control are indicated by *(p≤0.05). L: Summary graph of TUNEL+ mean densities of all oligodendroglia and other olig2+ glia subpopulations.

Dying oligodendroglia were indicated using the general cell death marker TUNEL [49] (Fig. 3A–G). In control ON, no dying NG2+/olig2– or NG2+/olig2+ cells were seen (Fig. 3H–I). Of the few TUNEL+ cells detected in control ON (4.9±1.7 cells/mm^2^), all were mature CC1+ oligodendrocytes (data not shown). As 61% of TUNEL+ cells were also mature NG2−/olig2+ cells, this population presumably encompassed the CC1+ population (Fig. 1A, 3J).

Following partial ON transection the total density of TUNEL+ cells markedly increased at 7 days (p = 0.003, 435.8±133.7 cells/mm^2^), 53.8% of TUNEL+ cells being oligodendroglia (data not shown). An increase in TUNEL+ cells occurred across all oligodendroglia populations at 7 days (p<0.05, Fig. 3H–L), however we found differences in the extent of cell loss in these subpopulations. A high proportion of NG2+/olig2– cells were TUNEL+ at 7 days (Fig. 3H), but the population density remained stable indicating that the proliferation observed within this population (Fig. 2K) was sufficient to maintain cell numbers. In contrast, NG2+/olig2+ cells proved vulnerable to secondary degeneration, with nearly 50% of NG2+/olig2+ cells positive for TUNEL at 7 days, and high proportions of TUNEL+ cells at 1 and 3 months (Fig. 3I). The death of NG2+/olig2+ cells was reflected in the sustained depletion of these cells from 7 days (p<0.05; Fig. 2L, 3I), suggesting that the proliferation of these cells was insufficient to maintain this population in ON exclusively vulnerable to secondary degeneration.

While a high proportion and density of TUNEL+ cells was seen at 7 days in NG2−/olig2+ cells, this resulted in no change in total cell density (Fig. 3J), suggesting this population was maintained by the observed proliferation (Fig. 2N). A key finding of this study was that mature CC1+ oligodendrocytes were resistant to secondary degeneration. Although the proportion of TUNEL+/CC1+ cells was significantly higher than control at 7 days (p<0.0001, 36.8±7.8 cells/mm^2^; Fig. 3K, L), the proportion of dying oligodendrocytes was very low at this time, and no change in the total density of these cells was observed following injury (Fig. 3K). Similarly, the combined densities of CC1+/olig2+ and CC1+/olig2– populations also remained unchanged following partial ON transection (Fig. 2O, P).

### Myelin Internodes under 110 µm Significantly Decreased during Chronic Secondary Degeneration

**Figure 4:**
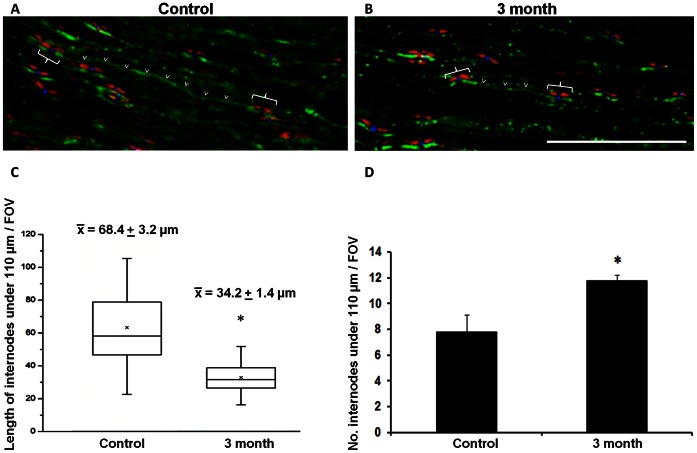
Myelin internode length following partial ON transection. Representative images of a single slice from the z stacks show ventral axons of control animals (A) and at 3 months following injury (B) anterogradely traced with CTB (green); paranodes are immunohistochemically labelled with Caspr and nodes with Nav1.6. C: The length of myelin internodes (indicated by <) under 110 µm were measured between paranodes (Caspr+ structures, red, confirmed by the presence of Nav1.6+ sodium channels, blue, at the node, indicated by brackets) and the data range, 25% and 75% percentile, median and mean (indicated by *) were displayed in the box plot. D: Mean number of internodes visible per FOV ± S.E (*p<0.05). Scale bar: 20 µm.

A defining characteristic of remyelination is the presence of shorter myelin internodes [13], [50], [51]. Here, we assessed internode length, using longitudinal ON sections and tracing CTB labelled axons between Caspr+ paranodes, surrounding Nav1.6+ nodes, at 3 months following partial ON transection. Previous studies have examined myelin internodes between adjacent paranodes in isolated whole tracts or single mechanically teased rubro-spinal tract fibers of contused spinal cord [51], [52]. However, as we were unable to tease apart axons of the optic nerve and the high density of labelled axons limited visualisation, only myelin internodes of 110 µm or less were quantified in the present study (Fig. 4). Given these caveats, we found that the length of myelin internodes under 110 µm significantly decreased at 3 months following partial transection, compared to control (p<0.0001), while the total number of myelin internodes under 110 µm detected within the field of view increased at 3 months (p = 0.039) (Fig. 4C, D). Given that we found no increase in the density of myelinating oligodendrocytes (CC1+/olig2+ or CC1+/olig2– cells) at 3 months (p>0.05), it is possible that during secondary degeneration individual oligodendrocytes are forming more internodes of shorter length.

## Discussion

To understand why myelin abnormalities persist during secondary degeneration [10], [11] we examined whether proliferation was sufficient to counter death of oligodendroglia in ventral ON exclusively vulnerable to secondary degeneration. Despite proliferation, death of NG2+/olig2+ progenitor cells contributed to the chronic loss of this population. Mature CC1+ cells remained unaffected following injury, indicating resistance to secondary degeneration *in vivo*, as has been reported in other models [53], [54]. There was an increase in CC1+/olig2– cells at 3 days that was indicative of oligogenesis, while the shortening of myelin internodes (less than 110 µm) at 3 months is suggestive of remyelination. Taken together, persistent myelin abnormalities characteristic of secondary degeneration may be due to the chronic loss of OPCs.

Other researchers have shown that proliferation of NG2+ cells increases at 2–7 days in residual tissue following chemically-induced demyelination and neurotrauma [55], [56]. Similarly, we found that OPCs proliferated within an acute window between 3 and 7 days in residual tissue vulnerable to secondary degeneration. The stimulus for OPC proliferation at 3 days was presumably an endogenous environmental cue other than the loss of mature CC1+ oligodendrocytes or demyelination. Although previous studies have shown that OPC proliferation is triggered by the loss of oligodendrocytes and demyelination [43], progenitor cells also proliferate in response to a non-demyelinating inflammatory lesion [57]. Therefore, OPCs vulnerable to secondary degeneration may proliferate in response to cytokines released by infiltrating microglia/macrophages and/or reactive astrocytes [58], known to increase at 3 days following partial ON transection [59]. While we have not directly assessed differentiation in the current study, the early increase in CC1+/olig2– cell density (3 days) was likely due to rapid differentiation of less mature populations (CC1+/olig2+ cells). Furthermore, it is possible that newly generated oligodendrocytes remyelinated axons, indicated by our finding of a subset of axons having shorter myelin internodes. In support of this, oligodendrocytes generated on the lesion border within the first week were involved in the chronic remyelination of axons, following spinal cord hemi-section [60]. In short, oligodendroglia respond to secondary degeneration, in that they proliferate, differentiate and likely remyelinate axons.

Despite having an early proliferative response, numbers of NG2+/olig2+ cells remained chronically decreased, suggesting that the adult ON did not have the capacity to completely restore the OPC population in ON exclusively vulnerable to secondary degeneration. Although OPCs are resistant to loss after repeated episodes of chemically-induced demyelination [61] and in MS [62], other studies have shown depletion in OPC numbers within residual tissue, following antibody-induced demyelination [63], indicating injury specific responses. Additionally, low densities of OPCs were reported in some lesion sites of MS patients, although these cells were quiescent and non-proliferating [64]. The chronic loss of OPCs during secondary degeneration may be attributed to: insufficient proliferation or symmetrical division of OPCs [65], differentiation into mature oligodendrocytes, or the observed sustained death of these cells. However, there was no chronic increase in overall CC1+ cell density (3 month) to reflect differentiation and subsequent oligogenesis at these later time points. Instead, death of OPCs likely contributed to the depletion of these cells during secondary degeneration. Previous *in vitro* studies have shown that OPCs are vulnerable to loss when exposed to oxygen/glucose deprivation [66], [67], oxidative stress [6], glutamate excitotoxicity [7] or hypoxia-ischemic injury [68]. In fact, a class of NG2+ cells involved in neuromodulation is particularly susceptible to glutamate excitotoxicity as these cells possess high numbers of glutamate receptors [20]. Taken together, the chronic reduction in OPCs was likely due to the inability of OPC proliferation to counter death of these cells during secondary degeneration.

Throughout adulthood, OPCs are responsible for myelinating axons that remained unmyelinated during development, or had ‘defective’/degenerated oligodendrocytes [35], [69]. The depletion of OPCs that we detected at 3 months may reduce adult myelinogenesis and replacement of ‘defective’ oligodendrocytes, seen in secondary degeneration [10]. OPCs also have a role in neuromodulation of nerve signaling [70], contacting the axon at the node of Ranvier [71] and engaging in rapid excitatory signaling and generation of action potentials at the node [20]. Indeed, developing oligodendrocytes do not remain in the CNS after proliferation if there is no axonal contact [72]. Therefore, chronic loss of OPCs may also disrupt neural signaling during secondary degeneration.

Strikingly, the mature CC1+ oligodendrocyte population appeared resistant to secondary degeneration *in vivo*. It is particularly interesting that no loss of mature oligodendrocytes was seen at 3 months following partial ON transection, given that we have previously reported a 30–37% decrease in axon density at this time, associated with retinal ganglion cell loss in ventral retina [10], [11]. This suggests that the same number of oligodendrocytes myelinate fewer axons. In support of our findings, no loss of mature oligodendrocytes was found in intact, residual tissue following spinal cord contusion [51], [53], although chronic demyelination has been observed in other CNS injury models [4]. Thus the loss of mature oligodendrocytes is not a characteristic feature of secondary degeneration. In conclusion, proliferation of OPCs is not sufficient to prevent death of these vulnerable cells. Furthermore, chronic loss of OPCs may contribute to myelin abnormalities and functional deficits during secondary degeneration.

## Materials and Methods

### Animals and Partial ON Transection Surgery

Ethics statement: all procedures were approved by The University of Western Australia Animal Ethics Committee. Adult female piebald-virol-glaxo (PVG) rats (150–200 g; 3 months old, Animal Resources Centre, Perth, Australia) were used. As described previously, rats were anaesthetised (Ilium xylazil and Ketamil I.P, Troy Laboratories) and a 200 µm incision (Radial Diamond Keratotomy knife, Geuder) made to the dorsal ON at 1 mm behind the right eye [8], [9]. Animals were euthanased (Euthal, I.P) at 1, 3, 7 days, 1 or 3 months following partial transection and experimental outcomes compared to control (normal, un-operated) right ONs (n = 5–8/group). An age-related decrease in the efficiency of remyelination has been demonstrated in aged rats that were 8–10 months older than controls [22]; it is not anticipated that the 3 month age difference between control and experimental animals is significant in the current study.

### Immunohistochemistry and Terminal Deoxynucleotidyl Transferase - Mediated dUTP Nick-end (TUNEL) Labelling

Animals were transcardially perfused and right ONs post-fixed consistently for exactly 24 hours. Parallel series of transverse sections (14 µm) were made and processed with the DeadEnd ™ Fluorometric TUNEL system (Invitrogen) according to the manufacturer’s instructions. Immunohistochemistry using antibodies to Ki67 involved an antigen-retrieval step in which slides were immersed in 10 mM sodium citrate in 0.05% Tween and heated to boiling point in the microwave for 30 seconds. Slides were then pre-blocked with 10% normal donkey serum (NDS) in 0.2% Triton-X in PBS for 20 minutes and incubated overnight at 4°C with primary antibodies (in 10% NDS, 0.2% Triton-X in PBS); rabbit anti-Ki67 (1∶200, Abcam), mouse anti-NG2 (1∶250, Invitrogen), rabbit anti-Olig2 (1∶500, Abcam), mouse anti-Nkx2.2 (1∶5, DSHB), rabbit anti-desmin (1∶200, Dako), rabbit-anti-MBP (1∶250, Millipore), mouse anti-CC1 (1∶500, Calbiochem), goat anti-Olig2 (1∶500, R & D Systems), goat anti-GFAP (1∶500, Sigma), rabbit anti-IBA1 (1∶500, Wako) or mouse anti-βIII tubulin (1∶750, Covance, used to assess the extent of the ON injury site). Following thorough washing, sections were incubated with species-appropriate secondary antibodies AlexaFluor® 488, AlexaFluor® 555, AlexaFluor® 647 (1∶400, 0.2% Triton-X in PBS, Invitrogen) and Hoescht (1∶1000, Invitrogen) for 1.5 hours at room temperature. Slides were mounted with ProLong® Gold (Invitrogen) and imaged using fluorescence microscopy.

### Fluorescence Microscopy, Image Analysis and Quantification of Oligodendroglia

For sections containing the injury site, fluorescence images (x 25 mag) of the entire ventral ON were generated using a Nikon Eclipse Ti inverted fluorescent microscope (Nikon Corporation, Japan) and imaging software package NIS Elements Advance Research. Images were taken in a series of z stacks (0.5 µm optical section thickness, range: 6 µm) and deconvoluted using NIS Elements software. In order to ensure optimal visualisation and true colocalisation of markers, a single image in the z plane (selected based on image clarity) was used to count all immunopositive oligodendroglia in ventral ON using the image analysis software ImageJ cell counter plugin, and data expressed per mm^2^ assessed.

### Anterograde Tracing and Myelin Internode Analysis

Animals (control n = 6; 3 months after injury: n = 5) were anaesthetised (as above), connective conjunctiva tissue cleared from the right eye and a Hamilton’s syringe used to deliver 3 µl of cholera toxin subunit B (Recombinant) AlexaFluor® 488 Conjugate (CTB, 0.5% in sterile deionised H_2_0, Invitrogen). Animals were transcardially perfused 3 days following CTB injection, ONs were post-fixed and longitudinal sections (14 µm) incubated overnight at 4°C with primary antibodies mouse anti-Caspr (1∶750, Abcam) and rabbit anti-Nav1.6 (1∶125, Alamone Laboratories) secondary antibody AlexaFluor® 546 or 647 and sections mounted with ProLong® Gold. Images of ON (Nikon Eclipse, NIS Elements software) directly below the injury site i.e. ventral ON, were taken in a series of z stacks (0.1 µm optical section thickness, range: 7 µm, covering a total field of view (FOV) of 0.05 mm^2^). Myelin internode lengths were measured by tracing axons from one paranode (Caspr+ structures, surrounding Nav1.6+ node) to another through the z stack using ImageJ, similar to that described in [51], [52]. The high density of CTB labelled axons limited visualisation of individual internodes, enabling only myelin internodes of 110 µm or less to be reliably quantified. As such, all detectable internodes of less than 110 µm in length were measured within the defined FOV, with a total of 39 internodes measured in control ON and 47 in ventral ON at 3 months after injury.

### Statistics

Data were analysed using the statistics program Origin (OriginLab Corporation, Microsoft License, Massachusetts, USA). Data were expressed as cells/mm^2^ ± S.E. All outcome measures were normally distributed with equal variances, and significant differences between control and experimental groups were determined using one-way ANOVA (p≤0.05) and Bonferroni/Dunn *post hoc* test.
